# The relationship between social capital and self-rated health: a multilevel analysis based on a poverty alleviation program in the Philippines

**DOI:** 10.1186/s12889-019-8013-5

**Published:** 2019-12-05

**Authors:** Natalee Hung, Lincoln Leehang Lau

**Affiliations:** 1 Department of Epidemiology, Biostatistics and Occupational Health, McGill University, Montreal, Quebec Canada; 2 Dalla Lana School of Public Health, University of Toronto, 155 College Street, Toronto, Ontario M5T 1P8 Canada; 3 International Care Ministries, Manila, Philippines

**Keywords:** Social capital, Self-rated health, Poverty alleviation, Philippines

## Abstract

**Background:**

Poor health is both a cause and consequence of poverty, and there is a growing body of evidence suggesting that social capital is an important factor for improving health in resource-poor settings. International Care Ministries (ICM) is a non-governmental organization in the Philippines that provides a poverty alleviation program called *Transform*. A core aim of the program is to foster social connectedness and to create a network of support within each community, primarily through consistent community-led small group discussions. The purpose of this research was to investigate the relationship between social capital and self-rated health and how ICM’s *Transform* program may have facilitated changes in those relationships.

**Methods:**

Three types of social capital were explored: bonding-structural, bridging-structural and cognitive. Using cross-sectional data collected before and after *Transform*, multilevel modelling was used to examine their effects on self-rated health between the two time points.

**Results:**

The analyses showed that while social capital had minimal effects on self-rated health before *Transform*, a series of associations were identified after the program. Evidence of interdependence between the different types of social capital was also observed: bonding social capital only had a beneficial effect on self-rated health *in the presence* of bridging social capital, but we found that there was a 17 percentage point increase in self-rated health when individuals possessed all possible bridging and bonding relationships. At the same time, our estimates showed that maximising all forms of social capital is not necessarily constructive, as the positive effect of cognitive social capital on self-rated health was weaker at higher levels of bridging social capital.

**Conclusions:**

The results from this study has shown that building social capital *can* influence the way people perceive their own health, which can be facilitated by intervention programs which seek to create bonding and bridging relationships. *Transform’s* intentional design to learn in community could be relevant to program planners as they develop and evaluate community-based programs, making adaptations as necessary to achieve organisation-specific goals while acknowledging the potential for varied effects when applied in different contexts or circumstances.

## Background

The cycle of poor health and poverty is difficult to break [1], and social factors have been identified to be at the root of this challenge. Marmot [2] explains that health is often beyond an individual’s physical state, but rather, a product of public policies that shape the social environments people are exposed to. As the significance of social determinants of health was promoted, the mechanisms through which relationships between people are translated into physical outcomes emerged as a key area for further research [3]. This is of particular importance in the analysis of resource-poor settings, especially with research claiming that the lack of human and financial capital in developing countries magnifies the influence of social capital on physical health [4].

Social capital is a concept that is based strongly on social relations, and a range of studies confirm that the strengthening of such is crucial to achieving desired development and poverty reduction outcomes [5–7]. Kawachi, Subramanian and Kim [8] echo this, stating that “a relationship between social capital and physical health have been more consistently found in societies with high levels of economic inequality, whereas the links are much weaker or non-existent in more egalitarian societies” [p.22]. This study aims to contribute to the growing research base that examines the intermediary effect of social capital on health as a key component in development interventions.

### ICM’s *Transform* program in the Philippines

The Philippines has seen robust economic growth in the last decade [9], and its economy continues to expand in the face of slowing global trade and investment flows [10]. Although it is one of the fastest-growing economies in Southeast Asia [10], poverty remains widespread with almost a quarter of the Philippine population living under the poverty line [11].

International Care Ministries (ICM) is an NGO that runs a 16-week poverty-alleviation program called *Transform* for populations in the Philippines living in ‘ultrapoverty’. Households that report to have a daily income of less than US$0.50 per person are classified as in ultrapoverty ([12]: p.2]). Among the participants ICM provided programs to in 2016, the average daily income per person was approximately US$0.28 ([13]: p.3]). *Transform* is carried out in numerous communities across the southern two-thirds of the Philippines, meaning that ICM serves a wide range of people living in diverse geographical landscapes with distinct cultures, who work in different trades and experience a variety of difficulties in the face of poverty. ICM has identified three key areas as the primary barriers to escaping poverty: a lack of relational skills, health knowledge and livelihood experience ([13]: p.5]), and a core aim of *Transform* is to foster social connectedness and to create a network of support within communities. Weekly Health and Livelihood training sessions are undertaken in large group settings, and the curriculum is designed to encourage participation in community-led small group work (of five or six) to discuss topics that were taught during the larger group sessions. There is also a Values curriculum dedicated to “fostering attitudes and behaviours that build strong relationships” ([13]: p.6]). To facilitate this, *Transform* is designed with four layers of support with different but complementary roles [13]:

1. The pastor, a known leader in the community, invites participants to join the program and runs the Values training;
2. Six volunteer counsellors from the local community help implement the program and provide encouragement;
3. Two ICM staff members from outside the community lead the Health and Livelihood training;
4. The 30 participants themselves learn and grow together.

Uphoff and Wijayartna [14] argue that all cultures have underlying norms, values, attitudes and beliefs that predispose cooperative behaviours, but its expression can be inhibited if appropriate forms of structural social capital are lacking in the communities. Through *Transform’s* weekly training sessions*,* community members had access to a new platform of social contact for 16 weeks through which relationships could be built. By investigating the outcomes of an intervention program with explicit aims to *create* social capital, our intention was to better understand its relationship with health in resource-poor settings as well as to evaluate the place for social capital in the design of health promotion interventions.

### Social capital

Modern developments of social capital that instigated widespread academic interest are credited to the seminal works of Pierre Bourdieu [15], James Coleman [16], and Robert Putnam [17]. Bourdieu and Coleman described social capital as another form of productive ‘capital’, while Putnam, in contrast, saw social capital less as a resource but shifted the definition to focus on qualities of social cohesion that may underlie these networks. If social cohesion refers to the extent of solidarity and connectedness in a society [18], Putnam’s social capital looked closer into these “features of social organisation, such as networks, norms, and trust, that facilitate coordination and cooperation for mutual benefit” ([17]: p. 167]). Given the context of ICM’s *Transform* program and its focus on fostering social connectedness, Putnam’s definition of social capital, being rooted in notions of social cohesion, was the most appropriate.

### Types of social capital

There are many different types of social capital, but one notable distinction is between structural and cognitive forms of social capital. Structural social capital refers to externally observable aspects of social organisation, such as roles, rules, procedures and precedents [14], for example, civic participation or group membership. Cognitive social capital is more internal and subjective, referring to shared norms, values, attitudes and beliefs [14]. Although they have been presented as mutually reinforcing components [14], it is nevertheless important to differentiate between these categories of social capital, as they can have different effects depending on both the individual’s characteristics and that of the community’s [19].

Another important conceptual development was the distinction between bonding and bridging social capital [20–22]. Bonding capital is accessed from relations between people in groups who share a social identity, for example, gender, class or ethnicity [8]. Bridging capital is accessed from relations that traverse boundaries of social identity [8], such as between people from different ethnic or occupational backgrounds. In Paxton’s [12] work on connected and isolated associational memberships, she categorised the connectedness of associations according to whether members have multiple memberships. Having associational networks that are linked to other voluntary associations, thus connected to the larger community, is reflective of *bridging* characteristics. Isolated memberships, in contrast, are “more dependent on close associates” ([12]: p.54]), reflecting *bonding* characteristics of fostering strong, in-group ties. Poortinga [23] proposed that they are characterised by different advantages: bonding relations are essential for social cohesion and support, whereas bridging relations build solidarity and respect amongst the wider community. Distinguishing between these two categories can, therefore, also help explain the occasionally inconsistent outcomes of social capital [8].

Critics have challenged the prevailing portrayal of social capital as inherently constructive. Putnam [18] warned that social inequalities may be embedded in social capital, as the ties that link members of a group together can also exclude other community members who do not share their social identity. Several studies have found that respondents who were less attached to their immediate community or who reported ties with people outside their social milieu displayed comparatively better health outcomes ([Mitchell and LaGory, 2002 and Caughy et al., 2003, as cited in [8]). The reliance of health improvement on the ability to access resources beyond the social boundaries of homogeneous circles suggest that community development efforts require *both* bonding and bridging social capital.

### Social capital interventions for health promotion

There is a general consensus that social capital cannot be easily created [24, 25]. In fact, Gugerty and Kremer [26] found that a project explicitly designed to strengthen social capital had no such effect. Building social capital requires a significant investment of time and resources, and dramatic results cannot be expected in the short-run. Murayama et al. [25] highlighted that social capital is heavily shaped by broad, structural forces, such as historical patterns of residential mobility or municipal investment in local infrastructure. As a result, the cultivation of social capital is often a complement to, rather than the chief end of, health promotion interventions.

**Fig. 1:**
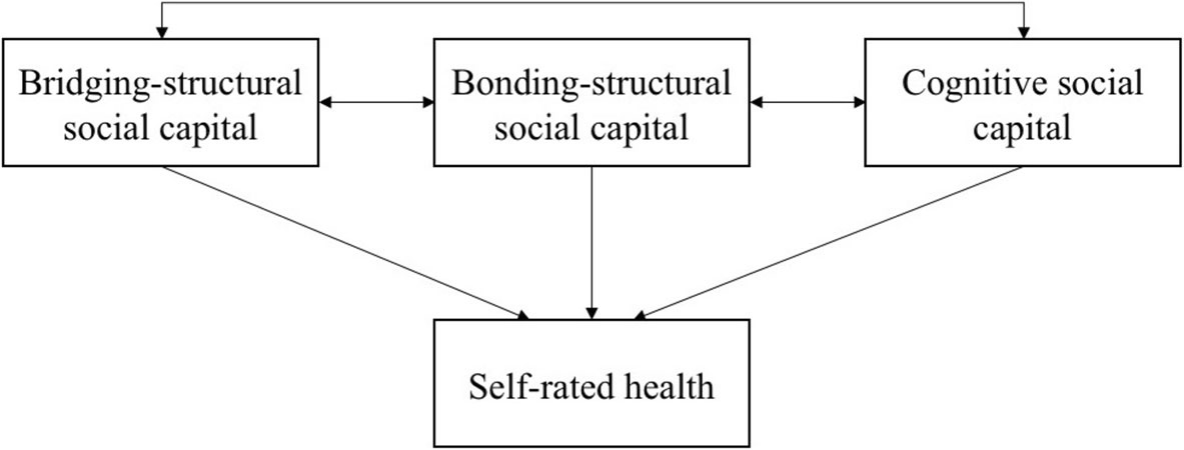
Schematic diagram illustrating the interdependence between bridging-structural social capital, bonding-structural social capital and cognitive social capital, and the potential pathways through which self-related health may be impacted

There is also surprising unity about the various mechanisms through which higher social capital can affect health: increased diffusion of information, provision of social and psychological support, ability to advocate for increased access to resources and enforcement of health-related behavioural norms through informal social control [19, 27–29]. De Silva and Harpham [30] phrased these pathways more simply: communities with greater social connectedness enable people to know more, feel, and act differently. In combination with a structured intervention program, this can instigate effects that further health promotion goals. In Fig. 1, we conceptualise the potential pathways by which the various types of social capital may impact self-related health.

The Medical Research Council’s guidelines, *Developing and Evaluating Complex Interventions* [31], highlights that a key question when evaluating complex interventions is to look at the “active ingredients” [p.7] within it and how they are exerting their effect. The aim is to “build a cumulative understanding of casual mechanisms, [to] design more effective interventions and apply them appropriately across group and setting” [p.7]*.* Our intention is to deepen our understanding of the context in which these mechanisms operate in with a view of informing future research in the design of complex interventions and subsequent evaluation approaches.

To guide our investigation, our primary research question was: What were the relationships between the different types of social capital and self-rated health before and after *Transform*, and how did those relationships change? As a sub-analysis, we also examined how the effects may be varied between communities. Although De Silva [32] claimed that the distinction between individual and ecological social capital is artificial, as “individuals’ social capital is influenced by what is available to them in the community, and the level of social capital in a community is determined by the social capital of its residents” [p.33], using a multilevel framework for analyses creates the “potential to account for group-level influences on individual health” ([8]: p.683]), and health determinants can be explored in acknowledgement of the *reciprocal* interactions between an individual and their contextual environment.

## Methods

### Data source

ICM regularly surveys *Transform* participants, and the following analysis was based on surveys conducted on participants who joined the program from October 2016 to January 2017. The baseline surveys were conducted as interviews by contract surveyors 2 weeks before the start of *Transform,* and the endline surveys were conducted 2 weeks after completion of the program. For this survey round, ICM purposively selected 50% of areas, representative of program implementation in the Central and Southern regions of the country. As a result, data was only available for 5 of the 10 ICM regional bases: Dumaguete, General Santos, Koronodal, Zamboanga Del Norte and Iloilo. Only participants that had *both* baseline and endline data were retained for analysis. The survey contained a range of individual-level items such as household composition, economic status, and physical and social well-being.

A codebook detailing the original survey questions used to operationalise the outcome and independent variables, as well as how they were coded, can be found in Additional file 1: Table S1A of the appendix.

### Outcome variable: self-rated health

ICM used EQ-5D as their general health measure [33]. EQ-5D consists of the EQ-5D descriptive system and the EQ visual analogue scale (EQ VAS). The EQ VAS is a quantitative measure of self-rated health. Participants were shown a scale and asked, “I would like to know how good or bad your health is TODAY. The scale is numbered from 0 to 100. 100 means the best health you can imagine. 0 means the worst case you can imagine. Please point to me where you are on the scale that indicates how your health is TODAY.” While self-rated health is a subjective assessment, EQ VAS is acknowledged as a valid measure of health status [34] and “a means of summarising overall health that is closer to the patient’s perspective” [35]. This was used as the outcome variable, operationalised as a continuous measure.

### Explanatory variables

#### Bonding-structural and bridging-structural social capital

Social capital is difficult to measure directly, so proxy indicators are often used to quantify its different dimensions [8]. To capture structural social capital, respondents were asked, “Do you belong to one of the following groups or associations?” and “Do you happen to personally know anyone who is a __________?” to identify group memberships and personal connections, respectively. Following Paxton’s [12] approach, these were operationalised into variables to represent bonding- and bridging-structural social capital for subsequent use in the analyses: for each type of group membership listed in the survey, we calculate the average number of total group memberships held using baseline data. Once the total number of group memberships were sorted in ascending order, those with a lower number of total group memberships were combined to represent ***bonding***-structural social capital, while those with a higher total were categorised as ***bridging***-structural social capital [36]. For example, individuals identifying membership in religious meetings (bonding) were found to, on average, have fewer total number of group memberships when compared to those who identified as belonging to barangay associations or savings groups (bridging). Barangays are the smallest local government unit in the Philippines. This procedure was repeated for types of personal connections.

Table 1 shows the categorisation of group memberships and personal connections as bonding or bridging social capital. In general, memberships or connections that were categorised as bonding are those that are easily accessible by the target population of *Transform*. By contrast, memberships or connections that were categorised as bridging, with differing levels of entry requirements, have additional dimensions of power and exclusion that make access by *Transform* participants more challenging. These baseline categorisations were kept consistent for the endline data analysis. Although some of these differentiations are country-specific and may not be generalisable, a similar process of differentiation can be replicated as appropriate for other contexts.

**Table 1:** Categorisation and description of group memberships and personal connections for operationalisation of bonding and bridging social capital

	Type of membership or connection	Categorisation	Description
*Group memberships*	Church	Bonding	The site of frequent community gatherings for church services, celebrations, festivals and holidays. Deeply intertwined with the cultural identity of Filipinos, as the Philippines is a religious country with the majority of their population self-identifying as Christian or Catholic [37].
	Religious meeting	Bonding	Additional informal religious gatherings, often organised within communities. A commonplace practice across the Philippines as a country with strong religious identifications.
	Barangay association	Bridging	Allows for participation in the smallest local government unit in the Philippines.
	Finance or credit group	Bridging	Requires members to surpass a minimum threshold of financial assets in order to gain entry.
	Savings group	Bridging	Requires members to surpass a minimum threshold of financial assets in order to gain entry. Thresholds are lower than that of finance or credit groups, but are less accessible as fewer have been established.
	Cooperative	Bridging	Local business organisations that are owned and controlled by a group people. Involvement requires sizeable assets and a business plan.
	Political association	Bridging	Involvement entails campaigning and taking part in local and national elections.
*Personal connections*	Pastor	Bonding	Protestant faith leaders (pastors) in the Philippines self-identify communities to work in. They typically reside directly in the community where the church is located, share similar demographic characteristics with the community members, and also occupy similar socioeconomic positions.
	Priest	Bridging	Catholic faith leaders (priests) in the Philippines are generally assigned to a larger geography (parish) that consists of multiple communities, which would include more individuals in their ‘service’ area than that of a pastor. As such, they may not come from the same background as those in the communities they serve.
	Barangay captain	Bonding	Local elected official representing the smallest government unit in the Philippines. They are usually from the barangay they represent and are well-known in their community.
	Barangay health worker	Bonding	Health-focused ‘volunteers’ that are recruited from local communities to be trained in front-line health service delivery.
	Health professional	Bridging	Doctors, nurse and mid-wives are scarce in rural areas. Trained health providers are mostly employed in urban centers and hospitals.
	Large business owner	Bridging	The target population of *Transform* (those living in extreme poverty) would have infrequent access to large business owners, who tend to occupy higher socioeconomic positions.
	Member of a co-op	Bridging	As proprietors of jointly-owned local enterprises, they are similar to large business owners, only the latter would be less commonly encountered.

The results of our calculations to differentiate bonding and bridging relationships can be found in Additional file 1: Tables S2A and S3A

To generate a summary score, the numbers of bonding and bridging relationships of each respondent were added up according to the categorisation in Table 1. The two sums were divided by the maximum number (5 for bonding, 9 for bridging) to get a standardised score between 0 and 1. These scores were used as continuous measures for bonding- and bridging-structural social capital.

#### Cognitive social capital

In this study, cognitive social capital measures were chosen to represent the expectations of how people may behave. The relevant questions in the survey asked respondents whether they generally perceived people as trustworthy (“In general, would you say that most people can be trusted or that most people cannot be trusted?), helpful (“Would you say that most of the time people try to be helpful, or that they are mostly just looking out for themselves?”) and fair (“Do you think most people would try to take advantage of you if they got a chance, or would they try to be fair?”). An additive score was derived from their binary responses and divided by the maximum number, yielding a standardised score between 0 and 1 for use as a continuous variable.

#### Socio-demographic variables

Only adults aged 18 and above were retained in the sample for analyses, with age used as a continuous measure. Other personal characteristics that were taken into account included gender and marital status as categorical variables. The number of people in the household, was included in the models as a continuous variable.

Two variables were used as proxies of economic position. Educational attainment captured in three categories: 1) none, 2) high school or less, and 3) college or more, and the respondent’s employment status, which was measured as a binary variable of whether they are in work or not. Income was not used because *Transform* is only delivered to households with a daily income of less than US$0.50.

Other variables included in the models were food security, which assessed the extent to which respondents had access to regular daily meals, and hygiene, specific to hand washing practices. Standardised scores between 0 and 1 were calculated for each and operationalised as continuous variables. Identification with religion, measured as a categorical variable, was also included. Types of religions considered in the study were Roman Catholic, Protestant, Muslim, Iglesia ni Cristo, or Others.

### Data analysis

Given that the communities that received *Transform* were situated across a wide range of geographical types, we used multilevel mixed-effects models to address our research question [38]. Every community that *Transform* is delivered to is assigned a unique identifying number. Cross-sectional models were generated separately for baseline and endline data so that the relationships between different types of social capital and self-rated health could be compared between the two time points.

In these multilevel models, individual participants formed level 1, and unique community ID was fitted as a random effect, forming level 2. Model 1 is the variance components (VC) model with no explanatory variables, and Model 2 is a random intercept (RI) model including all the socio-demographic, food security and hygiene, and social capital variables. Preliminary model explorations revealed that the full model has the most explanatory power, so the intermediary stages of cumulatively controlling for different variable groups was omitted. Model 3 allowed for cognitive social capital to have a different effect for each community. Given that, conceptually, the different constructs of social capital are closely related, we explored potential interactions between the social capital variables. Having found low correlation between them (See Additional file 1: Table S4A and S5A), interaction terms were added to Model 3 using a stepwise approach (See Additional file 2). As the most parsimonious model, Model 4, which included the interaction effects between bonding and bridging social capital and between bridging and cognitive social capital, was selected as the final model in the endline multilevel analysis.

The procedures described above were conducted using the statistical package Stata 15.1, and all figures were drawn using R version 3.4.3.

## Results

The study population consisted of *Transform* participants from 44 communities in 5 of the regional bases where ICM operates: Dumaguete, General Santos, Koronodal, Zamboanga Del Norte and Iloilo. Of the 2208 participants targeted to complete the questionnaire, 2166 were surveyed (98%). Table 2 shows characteristics of these participants who joined the program from October 2016 to January 2017. A large majority of participants were females who were married or living with a partner. The mean age was about 40 years old, and although most had at least completed high school, over half were not in work at the time the survey was undertaken. These characteristics are reflective of the demographic who tend to have the availability to participate in *Transform’s* training curriculum.

**Table 2:** Characteristics of *Transform* participants in the Central and Southern regions of the Philippines who joined the program from October 2016 to January 2017

Characteristics	Baseline	Endline
*Sample size (no.)*	2166	2166
*Number of communities (no.)* ^a^	44	44
*Mean age, years (no. (SD))*	39.80 (13.83)	40.38 (14.02)
*Sex (no. (%))*		
Male	169 (7.80)	174 (8.03)
Female	1997 (92.20)	1992 (91.97)
*Marital Status (no. (%))*		
Married	1507 (70.06)	1526 (70.52)
Live-in	433 (20.13)	442 (20.43)
Separated	39 (1.81)	28 (1.29)
Widowed	124 (5.76)	117 (5.41)
Single	48 (2.23)	51 (2.36)
*Number of people in household (no. (SD))*	4.61 (1.88)	4.73 (1.89)
*Highest educational attainment (no. (%))*		
None	79 (3.65)	90 (4.16)
High school or below	1919 (88.60)	1909 (88.13)
College or above	168 (7.76)	167 (7.71)
*Is the respondent in work? (no. (%))*		
No	1384 (63.90)	1331 (61.45)
Yes	782 (36.10)	835 (38.55)
*Religion (no. (%))*		
Roman Catholic	954 (48.77)	770 (39.90)
Protestant	816 (42.72)	946 (49.02)
Muslim	4 (0.20)	4 (0.21)
Iglesia ni Cristo	13 (0.66)	11 (0.57)
Other	169 (8.64)	199 (10.31)

^a^The analysis was conducted for the following regional bases where ICM operates: Dumaguete, General Santos, Koronodal, Zamboanga Del Norte and Iloilo

Only 1942 (87.9%) and 1928 (87.3%) cases were retained for the baseline and endline analyses, respectively, due to missing data.

### Baseline data

The results of multilevel regression on baseline data are displayed in Table 3. A likelihood ratio (LR) test (see Additional file 1: Table S9A) was conducted, resulting in a test statistic of 28.76 (*p* < 0.001), indicating that multilevel analysis was appropriate.

**Table 3:** Multilevel mixed-effects linear regression testing the impact of social capital on self-rated health, pre-*Transform* (Baseline data)

Variables	Unstandardised estimate (S.E.)	
	Model 1	Model 2
*Intercept*	80.55 (0.52)***	69.72 (3.74)***
*Fixed effects*		
Bonding SC		1.72 (1.84)
Bridging SC		−3.45 (2.44)
Cognitive SC		3.22 (1.11)**
*Random effects*		
*Level 1 variance*	216.22 (23.34)	168.69 (18.27)
*Level 2*		
Variance of random intercepts (RI)	6.48 (2.34)	5.45 (1.79)
ICC	0.029	0.031
*Model information criteria*		
AIC	17,836.24	15,551.43

N_i_ = 1942, N_j_ = 44

* *p* < 0.05; ** *p* < 0.01; *** *p* < 0.001

The following covariates were also controlled for in the models: age, sex, marital status, number of people in household, highest educational attainment, whether the respondent is in work, religion, food security and hygiene. The estimates for these variables can be found in Additional file 1: Table S6A

In Model 2, the significant results for age were consistent with expectations of deteriorating health with increasing age. This model also confirmed the importance of having stable access to food and better hygiene practices for improved self-rated health scores as both predictors were significant at *p* < 0.001 with likewise high estimates. Being separated compared to married, as well as having more people in a household, was associated with higher self-rated health, although the effect was weaker. Estimates for these covariates can be found in Additional file 1: Table S6A.

Finally, we looked more closely at the models with our key variables of interest in model 2. We saw that both bonding and bridging social capital were not significantly correlated to the outcome. It seems that Pre-*Transform*, self-rated health was not an area affected by those relationships. In contrast, cognitive social capital was highly significant at *p* < 0.01. No effects were observed with the inclusion of the interaction terms at baseline, so they were not retained in the model. The low intraclass correlation coefficient (ICC) figures across Table 3 indicated that these results were mostly explained by characteristics of the individual participants as opposed to the communities-level characteristics included in this model.

The ICC, calculated from the random part of Model 2, revealed that 3.1% of the variance in self-rated health was due to differences between communities, showing that the variation was mostly *within* communities.

### Endline data

The multilevel models on endline data is found in Table 4. The LR test statistic was 36.38 (*p* < 0.001), showing that a multilevel structure was appropriate. Individual variance appeared high, but the relative variation between-groups to within-groups in the VC model was 0.04. Variation was still mostly at the *individual* level, but compared to pre-*Transform*, the influence of participants’ communities on their self-rated health seemed to be slightly stronger after partaking in the program.

**Table 4:** Multilevel mixed-effects linear regression testing the impact of social capital on self-rated health, post-*Transform* (Endline data)

Variables	Unstandardised estimate (S.E.)			
	Model 1	Model 2	Model 3	Model 4
*Intercept*	79.71 (0.74)***	31.81 (10.02)***	31.78 (5.53)***	34.31 (10.12)**
*Fixed effects*				
Bonding SC		−4.44 (3.31)	−3.25 (2.24)	−10.15 (4.20)*
Bridging SC		6.20 (3.16)*	5.38 (2.19)*	−7.80 (12.46)
Cognitive SC		4.22 (2.66)	5.42 (2.77)*	10.14 (4.12)*
Bonding SC x Bridging SC				27.26 (13.82)*
Bridging SC x Cognitive SC				−13.94 (6.99)*
*Random effects*				
*Level 1 variance*	375.87 (37.90)	361.71 (36.09)	326.28 (10.76)	323.96 (31.17)
*Level 2*				
Variance of random intercepts (RI)	14.82 (5.89)	15.65 (6.21)	196.60 (53.09)	195.74 (72.06)
Variance of random slopes (RS)			256.37 (71.25)	256.41 (82.10)
Covariance between the RI and RS			−216.55 (59.85)	− 215.56 (75.40)
ICC^a^	0.038	0.041		
*Model information criteria*				
AIC	19,041.18	16,917.78	16,796.48	16,788.62

* *p* < 0.05; ** *p* < 0.01; *** *p* < 0.001

^a^ A single estimate for ICC is not available for Models 3 and 4 as it is a function of the variable for which random slopes are specified (i.e. conditional on the values of cognitive social capital)

The following covariates were also controlled for in the models: age, sex, marital status, number of people in household, highest educational attainment, whether the respondent is in work, religion, food security and hygiene. The estimates for these variables can be found in Additional file 1: Table S7A

Variables that were strong and significant predictors in the baseline analysis remained so in the endline analysis: age, food security and hygiene. While the coefficients for age only fell by around 0.1, compared to baseline, the coefficients for food security and hygiene more than doubled in all models. This may be a reflection of the efficacy of *Transform’s* Health and Livelihood training in improving hygiene practices and access to food. Estimates for these covariates can be found in Additional file 1: Table S7A.

The effect of all social capital variables on self-rated health were not present in the full multilevel RI model, but when we allowed the slopes to vary on cognitive social capital in the random slopes (RS) model, the estimates for bridging and cognitive social capital became statistically significant. With the addition of the two interaction effects in Model 4, however, it was bonding and cognitive social capital that were significant, and we also observed substantial movement in all the social capital estimates. The LR test confirmed that this was a better fit for the data, compared to Model 3, with a test statistic of 11.85 (*p* < 0.001). We outline the effects of each interaction term in turn.

Taking into account the interaction effect between bonding and bridging social capital, an individual who possessed all possible bridging relationships would have experienced an increase of 17.11 in self-rated health for an additional increment of bonding social capital. The negative bonding social capital estimate and the positive interaction term of comparatively greater magnitude implied that bonding social capital only had a beneficial effect on self-rated health *in the presence* of bridging social capital. However, this would only be the case when an individual possessed enough bridging social capital for the interaction term to offset the negative effect of bonding social capital on its own. Bridging social capital could be interpreted similarly, but as its estimate was not statistically significant, we inferred that it had no independent effect on self-rated health.

The interaction effect between bridging and cognitive social capital was the reverse. The negative interaction term indicated that the positive effect of cognitive social capital on self-rated health was weaker at higher levels of bridging social capital, although this positive effect is only countered when a certain threshold of bridging social capital is surpassed.

**Fig. 2:**
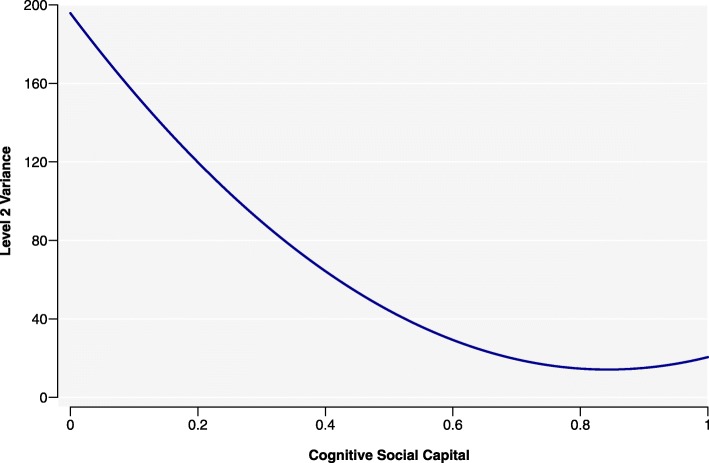
Variance of endline self-rated health scores between the 44 communities in which *Transform* was undertaken, by level of cognitive social capital. These communities were located in the Central and Southern regions of the Philippines. Decreasing level 2 variance is observed with higher cognitive social capital values, showing that participants in communities with higher cognitive social capital were more similar in their self-rated health score, compared to in communities with lower cognitive social capital

**Fig. 3:**
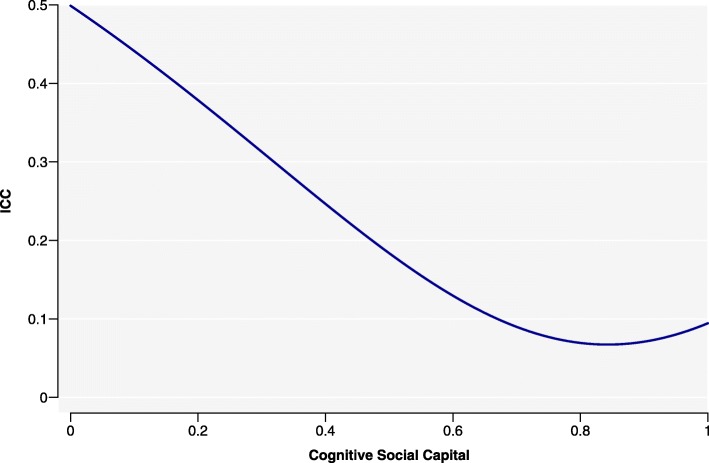
Percentage variance in endline self-rated health scores explained by differences between the 44 communities in which *Transform* was undertaken, by level of cognitive social capital. These communities were located in the Central and Southern regions of the Philippines. Self-rated health scores were more varied in communities with low cognitive social capital, with a greater proportion of unexplained variation attributable to community differences

Fitting a model with varying slopes means that level 2 variance was dependent on values of cognitive social capital (Fig. 2), and showed decreasing level 2 variance at higher cognitive social capital values. People in communities with higher cognitive social capital were more similar in their self-rated health score, compared to communities with lower cognitive social capital. Using level 2 variance to calculate ICC, we saw a similar output (Fig. 3). ICC was greater for low levels of cognitive social capital and smaller for higher levels. In other words, in communities with lower levels of cognitive social capital, a greater proportion of the unexplained difference in self-rated health scores between people was due to community differences. Although cognitive social capital was already a significant variable in the baseline analysis, allowing its slopes to differ did not improve the model. Only after the program did we see differences across communities in the relationship between participants’ perception of people as trustworthy, helpful and fair with self-rated health.

## Discussion

### Structural social capital

This analysis has yielded interesting findings about the interdependent nature of the two types of structural social capital and the way they influence self-rated health. Firstly, we note that effects were not observed in the baseline analysis. Bonding relationships *became* valuable for the promotion of self-rated health after participants had completed *Transform,* but only when the interaction term between bonding and bridging social capital was included in the model. Varying stances on the possible effects of bonding social capital exist in literature. Some have suggested that it is beneficial for social cohesion and support [23], but others have also suggested that it fosters inward-focused behaviour that is obstructive to health promotion [39]. The preliminary evidence in our study suggests that *both* views are plausible. While having links with people whom participants shared a social identify with was beneficial for self-rated health, this was only the case when they *also* knew well-connected people or were part of well-connected groups, as the results showed that being too exclusive had the opposite effect. This supports discussions that suggest health improvement in individuals is reliant on strong, in-group ties *as well as* the ability to access resources beyond the social boundaries of homogeneous circles ([Mitchell and LaGory, 2002 and Caughy et al., 2003, as cited in [8]). If we are to comprehensively understand the potential effects of bonding social capital, it should not be examined in isolation.

The picture for bridging social capital, however, was different. As stated in the introduction, *Transform* was itself a platform of structural social capital with the intention of facilitating the development of relationships with highly-connected groups and people. While its presence was a prerequisite for the positive effects of bonding social capital to materialize, on its own, bridging social capital did not appear to be associated with self-rated health. It is plausible that having access to certain information or experiences from ‘outside sources’ [40] is of limited value when these are not shared amongst people within the individual’s social milieu, rendering a null effect on self-rated health.

### Cognitive social capital

Our results have also demonstrated the important relationship between cognitive social capital and self-rated health. It was the only social capital variable that was statistically significant *both* before and after *Transform,* echoing existing empirical literature that higher levels of cognitive social capital, in particular, is associated with higher self-rated health [41, 42]. However, our analysis also revealed that after *Transform*, its effects were dependent on bridging social capital. Where higher levels of bridging social capital were observed, the beneficial effect of the cognitive social capital on health was weakened, perhaps owing to divergent norms, values and beliefs between heterogeneous groups.

We see that there is ambiguity when assessing the implications of forming relationships with heterogeneous groups: while bridging social capital was necessary for bonding social capital to enhance self-rated health, having ‘too many’ bridging relationships was not conducive to the role of cognitive social capital in supporting health improvements. This lends itself to the idea that higher levels of social capital are not necessarily health enhancing in every context*,* confirming challenges to its portrayal as inherently constructive.

When we examined the *contextual* effects of communities in the endline analysis, there was evidence of mutuality or interdependence in cognitive social capital levels within them: Fig. 2, which depicted level 2 variance from the RS model, revealed that there were greater similarities in self-rated health scores in communities with higher cognitive social capital. By contrast, in communities with low cognitive social capital, self-rated health scores were more varied, with a greater proportion of the variation being unexplained. We can infer that cognitive social capital had different effects on self-rated health in different communities; it was beneficial to participants with already-high levels of cognitive social capital, but for those with low levels, its favourable effect on self-rated health seemed to be overpowered by other factors that are not accounted for in the models.

It is also interesting that the community effects were only apparent in the endline analysis. While the data could not give us information about interactions that happened *during* the program, we can speculate people within the same community shared experiences that fostered perceptions of each other as trustworthy, helpful and fair in similar ways. This would reflect earlier studies that have suggested that the establishment of trust and norms of cooperation is built upon reciprocal exchanges between neighbours [Tempkin and Rohe, 1997, as cited in 20]. We must note that *Transform* was not designed to affect wider community trust. As a 16-week program for 30 participants, there were no expectations that perceptions regarding overall trustworthiness of the community would substantially improve (or otherwise change) over the course of the program. Nevertheless, we can observe applications for interventions that do address wider community safety: these results show that when programs are able to promote a sense of trustworthiness and security, for members of the community, these outcomes may be manifested in improved self-perceived health.

### Changing dynamics between social capital and self-rated health

Framing the relevance of strengthening social capital for intervention programs requires discussing the implications of the changing dynamics between social capital and self-rated health, supported by *Transform* as a training program and a socialisation platform.

Before *Transform*, structural social capital was not associated with self-rated health, and absence of interaction effects between the different types suggested that the mechanisms through which they seemed to operate were very much separate. With the sharing of challenges and learning experiences over the 16 weeks of training between the participants, their newly formed or strengthened relationships played a greater role in determining their self-perceived health status after the program. The *way* in which these different types of social capital facilitated self-rated health became interdependent. Bonding relations required bridging relations to support improvements in self-rated health, suggesting it is the *combination* of in-group social support *and* access to out-group perspectives and resources that is favourable to community development efforts. The building of such connections may not have transpired as such without *Transform.* If social influences are key in the determination of individual behaviour [43], the merit in undergoing growth *as a collective* is important to consider in the development of a community-based program with long-lasting outcomes.

We must also, however, be conscious that the nature of social capital is not one-dimensional. The combined effect of bridging social capital with cognitive social capital, by contrast, had the potential to be harmful to self-rated health, demonstrating that “the nature of social capital cannot be established once and for all as a positive or negative character for society unless we contextualise it” ([44]: p.604). This implies that the evidence about the effect of social capital on health *is* mixed, but this is to be expected when examining complex social interventions that act on complex social systems. Nevertheless, we believe that reporting these relationships and highlighting the patterns that exist between social capital and health is important to widen the evidence base, from which researchers may draw from, for future program planning and impact evaluations.

### Limitations of the study

There were a number of limitations of this study that must be acknowledged. Firstly, we consider the reliability of our key variables. While it could be argued that self-rated health is a subjective measure that cannot be an accurate reflection of ‘true’ health, studies have found that self-rated health is a strong predictor of mortality [45], showing that it *can* be representative of latent health status. Similarly, it is also difficult, if not impossible, to measure social capital *directly.* While proxies are the next best alternatives and are used by most empirical studies, the present one included, we must acknowledge that our findings are only applicable to the dimensions of social capital measured by the survey.

This study was cross-sectional in nature, so we were unable to determine the temporal relationship between social capital and self-rated health. There remains the possibility of reverse causality, where better self-rated health may have facilitated an increase in social capital, especially as participants received Health training as part of *Transform’s* Values, Health and Livelihood curriculum.

We realise that the nature of the data cannot give causal evidence for impact of the intervention on the stated outcomes. However, even the common perception that randomised controlled trials, as the gold standard, “always provide the strongest evidence for causality and for effectiveness” has been challenged [46]. Deaton and Cartwright instead advocate for an increased focus on the cumulative scientific process, highlighting the value of incorporating different methods to discover ‘why things work’ [46]. We still fully acknowledge that this study cannot elucidate the impact of *Transform*, nor does it attempt to. What it does give, however, are patterns of correlation over time, which we have stated as the intention of our investigations.

Lastly, some scholars believe that quantitative methods are inadequate for studying social processes, as they do not capture “social complexities, context or meaning” to the same extent as qualitative approaches ([Forbes and Wainwright, 2001, as cited in [23]). Quantitative methods are, however, better suited for capturing general trends across a range of communities, and this overview could be beneficial for narrowing the scope when designing future (qualitative) evaluative studies of *Transform*.

## Conclusion

The results from this study has shown that building social capital *can* influence the way people perceive their own health, and that there are ways for intervention programs to facilitate the creation of bonding and bridging relationships as forces that are mutually reinforcing. Embedded into the structure of *Transform’s* training sessions were small group interactions and discussions, as well as active efforts to coordinate visits from figures participants may not have otherwise come into contact with, such as local government officials, barangay captains and barangay health workers. *Transform’s* intentional design to learn in community could be relevant to program planners as they develop community-based programs, adapting it as necessary to achieve organisation-specific goals and acknowledging the potential for varied effects when applied in different contexts or circumstances. By demonstrating the varying mechanisms through which social capital may affect health across different groups and settings, this study can also inform improvements to evaluation approaches of complex interventions.

Our analyses of cognitive social capital reinforce the idea that the specific effects of social capital are context-dependent and likely heterogeneous across communities. We may postulate that for isolated and dispersed communities, where there are less opportunities for connections and relationships to form or deepen, an intervention that incorporates the building of social capital into its operations could be particularly valuable to the community members and their health. It is also important to acknowledge that the main driving force behind the outcomes of an intervention program will be the broader structural forces of society, and social capital should be valued as a complement, rather than the chief end, of intervention objectives.

## Supplementary information

**Additional file 1.** Appendices. Supplementary tables.

**Additional file 2.** Multilevel mixed-effects linear regression testing the impact of social capital on self-rated health, post-Transform. Supplementary table showing model building with social capital interaction terms

## Data Availability

The datasets analysed during the current study are available in the Mendeley Data repository, 10.17632/h39dytfg2v.1

## Abbreviations

EQ VAS: EQ Visual Analogue Scale
ICC: Intraclass Correlation Coefficient
ICM: International Care Ministries
LR: Likelihood Ratio
RI: Random Intercept
RS: Random Slopes
VC: Variance Components
