# Supplementary material for: The relationship between social capital and self-rated health: a multilevel analysis based on a poverty alleviation program in the Philippines
Source: BMC Public Health. 2019 Dec 5;19:1641. doi: 10.1186/s12889-019-8013-5 (PMC6896750; doi:10.1186/s12889-019-8013-5)
Supplement: Supplementary file 1 — Additional file 1. Appendices. Supplementary tables. [file 12889_2019_8013_MOESM1_ESM.docx]

**Appendices (Supplementary)**

| Table 1A. Codebook detailing variables used in the analyses: variable name, question wording in the survey, variable type and how they were coded | | | | | |
| --- | --- | --- | --- | --- | --- |
| **Level** | **Item** | **Variable name †** | **Description** | **Variable Type** | **Values** |
| Outcome | EQ VAS | eqvas | Self-rated health operationalised by the question: "I would like to know how good or bad your health is TODAY. The scale is numbered from 0 to 100. 100 means the best health you can imagine. 0 means the worst case you can imagine." | Continuous | 0 to 100 |
| Individual level | Bonding-structural social capital | bonding | Standardised score of belonging to groups or having personal connections categorised as bonding (low connectedness), derived from binary responses to the following questions:  "Do you belong to one of the following groups or associations?" *Bonding group memberships:* Church and religious meeting.  "Do you happen to personally know anyone who is a __________?" *Bonding personal connections:* Barangay captain, pastor and barangay health worker | Continuous | 0 (Low) to  1 (High) |
|  | Bridging-structural social capital | bridging | Standardised score of belonging to groups or having personal connections categorised as bridging high connectedness), derived from binary responses to the following questions:  "Do you belong to one of the following groups or associations?" *Bridging group memberships:* Barangay association, finance or credit group, savings group, cooperative, political association.  "Do you happen to personally know anyone who is a __________?" *Bridging personal connections:* Health professional, priest, large business owner, member of a co-op. | Continuous | 0 (Low) to  1 (High) |
|  | Cognitive social capital | cognitive | Standardised score of binary responses to the following questions: "In general, would you say that most people can be trusted or that most people cannot be trusted?" "Do you think most people would try to take advantage of you if they got a chance, or would they try to be fair?" "Would you say that most of the time people try to be helpful, or that they are mostly just looking out for themselves?" | Continuous | 0 (Low) to  1 (High) |
|  | Age | age | Age of the respondent | Continuous | ≥18 |
|  | Sex | sex | Sex of the respondent | Categorical | 2 = Female 1 = Male |
|  | Marital status | marstat | Marital status of the respondent | Categorical | 5 = Single 4 = Widowed 3 = Separated 2 = Live-in 1 = Married |
|  | Number of people in the household | hhnum | Number of people who usually sleep and eat in the participant's household | Continuous | ≥1 |
|  | Educational attainment | edu | Respondent's highest level of schooling completed | Categorical | 2 = College or higher 1 = High school or below 0 = None |
|  | Employment status | work | Whether the respondent is currently in work | Categorical | 1 = Yes 0 = No |
|  | Food security | foodsec | Standardised score of ordinal responses to the following questions: "Over the last MONTH, did your household (have)... No food to eat of any kind in your household? Go to sleep at night hungry? Go a whole day and night without eating?" | Continuous | 0 (Low) to  1 (High) |
|  | Hygiene | hygiene | Standardised score of ordinal responses to the following questions regarding handwashing practices: "How often do you wash your hands?" "Do you have soap in your house?" "Do all members of the household wash their hands with ash or soap after using the latrine?" | Continuous | 0 (Low) to  1 (High) |
|  | Religious identification | religion | What is your current religion? | Categorical | 5 = Other 4 = Iglesia ni Cristo 3 = Muslim 2 = Protestant 1 = Roman Catholic |
| Community level | Community ID | cid | Unique identifying number of community where the respondent received *Transform* | Categorical | 1 to 44 |
| **†** Suffix "_pre" used for baseline data; suffix "_post" used for endline data | | | |  |  |

| Table 2A. Mean number of memberships types held by *Transform* participants, by group membership, calculated to differentiate bonding and bridging relationships | |
| --- | --- |
| **Type of group** | **Mean number of membership types for members of…** |
| *Bonding* |  |
| Church | 2.12 |
| Religious meeting | 2.66 |
| *Bridging* |  |
| Barangay association | 3.15 |
| Finance or credit group | 3.37 |
| Savings group | 3.55 |
| Cooperative | 3.72 |
| Political association | 4.04 |

| Table 3A. Mean number of connection types held by *Transform* participants, by personal connection, calculated to differentiate bonding and bridging relationships | |
| --- | --- |
| **Type of connection** | **Mean number of connection types for people who know…** |
| *Bonding* |  |
| Barangay captain | 3.88 |
| Pastor | 3.94 |
| Barangay health worker | 4.13 |
| *Bridging* |  |
| Health professional | 4.46 |
| Priest | 4.63 |
| Large business owner | 5.32 |
| Member of a co-op | 5.33 |

| Table 4A. Correlation between social capital variables at baseline | | | |
| --- | --- | --- | --- |
|  | **Bonding SC** | **Bridging SC** | **Cognitive SC** |
| **Bonding SC** | 1.00 |  |  |
| **Bridging SC** | 0.21 | 1.00 |  |
| **Cognitive SC** | 0.13 | 0.00 | 1.00 |
|  |  |  |  |
| Table 5A. Correlation between social capital variables at endline | | | |
|  | **Bonding SC** | **Bridging SC** | **Cognitive SC** |
| **Bonding SC** | 1.00 |  |  |
| **Bridging SC** | 0.25 | 1.00 |  |
| **Cognitive SC** | 0.09 | -0.01 | 1.00 |

| \| Table 6A. Multilevel mixed-effects linear regression testing the impact of social capital on self-rated health, pre-*Transform* (Baseline data) \| \| \| \| \| --- \| --- \| --- \| --- \| \| Variables \| Unstandardised estimate (S.E.) \| \| \| \|  \| Model 1 \| Model 2 \| Model 3 \| \| *Fixed effects* \|  \|  \|  \| \| Bonding SC \|  \| 1.72 (1.84) \| 1.77 (2.41) \| \| Bridging SC \|  \| -3.45 (2.44) \| -3.18 (8.26) \| \| Cognitive SC \|  \| 3.22 (1.11)** \| 3.22 (1.11)** \| \|  \|  \|  \|  \| \| Age \|  \| -0.28 (0.03)*** \| -0.28 (0.03)*** \| \|  \|  \|  \|  \| \| *Sex* \|  \|  \|  \| \| Male \| *- reference category -* \| \| \| \| Female \|  \| -0.48 (1.09) \| -0.48 (1.09) \| \|  \|  \|  \|  \| \| *Marital Status* \|  \|  \|  \| \| Married \| *- reference category -* \| \| \| \| Live-in \|  \| -1.35 (0.93) \| -1.35 (0.93) \| \| Separated \|  \| -4.35 (1.97)* \| -4.35 (1.97)* \| \| Widowed \|  \| 0.01 (1.17) \| 0.01 (1.17) \| \| Single \|  \| -2.58 (2.68) \| -2.58 (2.68) \| \|  \|  \|  \|  \| \| *Number of people in household* \|  \| 0.41 (0.17)* \| 0.41 (0.17)* \| \|  \|  \|  \|  \| \| *Highest educational attainment* \|  \|  \|  \| \| None \| *- reference category -* \| \| \| \| High school or below \|  \| -1.71 (1.45) \| -1.71 (1.46) \| \| College or above \|  \| 1.26 (1.51) \| 1.26 (1.52) \| \|  \|  \|  \|  \| \| *Is the respondent in work?* \|  \|  \|  \| \| No \| *- reference category -* \| \| \| \| Yes \|  \| 1.48 (0.81) \| 1.48 (0.81) \| \|  \|  \|  \|  \| \| *Religion* \|  \|  \|  \| \| Roman Catholic \| *- reference category -* \| \| \| \| Protestant \|  \| 0.70 (0.81) \| 0.70 (0.81) \| \| Muslim \|  \| 0.73 (3.24) \| 0.73 (3.26) \| \| Iglesia ni Cristo \|  \| -0.85 (4.37) \| -0.85 (4.36) \| \| Other \|  \| -0.53 (1.27) \| -0.53 (1.27) \| \|  \|  \|  \|  \| \| *Food security* \|  \| 11.23 (3.21)*** \| 11.23 (3.21)*** \| \| *Hygiene* \|  \| 5.46 (1.86)** \| 5.46 (1.86)** \| \|  \|  \|  \|  \| \| *Intercept* \| 80.55 (0.52)*** \| 69.72 (3.74)*** \| 69.67 (3.89)*** \| \|  \|  \|  \|  \| \| *Random effects* \|  \|  \|  \| \| *Level 1 variance* \| 216.22 (23.34) \| 168.69 (18.27) \| 168.69 (18.27) \| \| *Level 2* \|  \|  \|  \| \| Variance of random intercepts (RI) \| 6.48 (2.34) \| 5.45 (1.79) \| 5.45 (1.79) \| \|  \|  \|  \|  \| \| ICC \| 0.029 \| 0.031 \| 0.031 \| \|  \|  \|  \|  \| \| *Model information criteria* \|  \|  \|  \| \| -2 log-likelihood \| -8915.12 \| -7753.72 \| -7753.72 \| \| AIC \| 17836.24 \| 15551.43 \| 15553.43 \| \| N_i_ = 1942, N_j_ = 44 \| \|  \|  \| \| * p < 0.05; ** p < 0.01; *** p < 0.001 \|  \|  \|  \|  \| Table 7A. Multilevel mixed-effects linear regression testing the impact of social capital on self-rated health, post-*Transform* (Endline data) \| \| \| \| \| \| --- \| --- \| --- \| --- \| --- \| \| Variables \| Unstandardised estimate (S.E.) \| \| \| \| \|  \| Model 1 \| Model 2 \| Model 3 \| Model 4 \| \| *Fixed effects* \|  \|  \|  \|  \| \| Bonding SC \|  \| -4.44 (3.31) \| -3.25 (2.24) \| -10.15 (4.20)* \| \| Bridging SC \|  \| 6.20 (3.16)* \| 5.38 (2.19)* \| -7.80 (12.46) \| \| Cognitive SC \|  \| 4.22 (2.66) \| 5.42 (2.77)* \| 10.14 (4.12)* \| \|  \|  \|  \|  \|  \| \| Bonding SC x Bridging SC \|  \|  \|  \| 27.26 (13.82)* \| \| Bonding SC x Cognitive SC \|  \|  \|  \|  \| \| Bridging SC x Cognitive SC \|  \|  \|  \| -13.94 (6.99)* \| \|  \|  \|  \|  \|  \| \| Age \|  \| -0.15 (0.04)*** \| -0.15 (0.04)*** \| -0.15 (0.04)*** \| \|  \|  \|  \|  \|  \| \| *Sex* \|  \|  \|  \|  \| \| Male \|  \| *- reference category -* \| \| \| \| Female \|  \| -0.04 (2.27) \| -0.21 (1.66) \| -0.11 (2.16) \| \|  \|  \|  \|  \|  \| \| *Marital Status* \|  \|  \|  \|  \| \| Married \|  \| *- reference category -* \| \| \| \| Live-in \|  \| -1.81 (1.37) \| -1.69 (1.13) \| -1.55 (1.40) \| \| Separated \|  \| -1.56 (2.85) \| -1.72 (3.66) \| -1.31 (3.06) \| \| Widowed \|  \| -4.88 (2.85) \| -3.71 (2.04) \| -3.77 (2.50) \| \| Single \|  \| -0.61 (3.28) \| -1.23 (2.90) \| -1.09 (3.00) \| \|  \|  \|  \|  \|  \| \| *Number of people in household* \|  \| 0.18 (0.21) \| 0.13 (0.24) \| 0.13 (0.20) \| \|  \|  \|  \|  \|  \| \| *Highest educational attainment* \|  \|  \|  \|  \| \| None \|  \| *- reference category -* \| \| \| \| High school or below \|  \| 2.53 (3.88) \| 3.54 (2.22) \| 3.63 (3.85) \| \| College or above \|  \| 4.30 (4.26) \| 5.81 (2.71)* \| 5.74 (4.24) \| \|  \|  \|  \|  \|  \| \| *Is the respondent in work?* \|  \|  \|  \|  \| \| No \|  \| *- reference category -* \| \| \| \| Yes \|  \| 0.64 (1.27) \| 0.68 (0.93) \| 0.76 (1.17) \| \|  \|  \|  \|  \|  \| \| *Religion* \|  \|  \|  \|  \| \| Roman Catholic \|  \| *- reference category -* \| \| \| \| Protestant \|  \| 2.59 (1.22)* \| 2.98 (0.96)** \| 2.68 (1.16)* \| \| Muslim \|  \| 7.93 (4.02)* \| 6.95 (9.19) \| 6.66 (2.05)*** \| \| Iglesia ni Cristo \|  \| 9.24 (1.63)*** \| 7.07 (5.60) \| 7.16 (2.55)** \| \| Other \|  \| -0.98 (2.67) \| 1.02 (1.54) \| 1.19 (1.82) \| \|  \|  \|  \|  \|  \| \| *Food security* \|  \| 30.00 (8.72)*** \| 27.04 (5.23)*** \| 26.18 (8.92)** \| \| *Hygiene* \|  \| 19.27 (3.10)*** \| 19.41 (2.54)*** \| 19.77 (2.82)*** \| \|  \|  \|  \|  \|  \| \| *Intercept* \| 79.71 (0.74)*** \| 31.81 (10.02)*** \| 31.78 (5.53)*** \| 34.31 (10.12)** \| \|  \|  \|  \|  \|  \| \| *Random effects* \|  \|  \|  \|  \| \| *Level 1 variance* \| 375.87 (37.90) \| 361.71 (36.09) \| 326.28 (10.76) \| 323.96 (31.17) \| \| *Level 2* \|  \|  \|  \|  \| \| Variance of random intercepts (RI) \| 14.82 (5.89) \| 15.65 (6.21) \| 196.60 (53.09) \| 195.74 (72.06) \| \| Variance of random slopes (RS) \|  \|  \| 256.37 (71.25) \| 256.41 (82.10) \| \| Covariance between the RI and RS \|  \|  \| -216.55 (59.85) \| -215.56 (75.40) \| \|  \|  \|  \|  \|  \| \| ICC \| 0.038 \| 0.041 \|  \|  \| \|  \|  \|  \|  \|  \| \| *Model information criteria* \|  \|  \|  \|  \| \| -2 log-likelihood \| -9517.59 \| -8436.89 \| -8374.24 \| -8368.31 \| \| AIC \| 19041.18 \| 16917.78 \| 16796.48 \| 16788.62 \| \| N_i_ = 1928, N_j_ = 44 \| \|  \|  \|  \| \| * p < 0.05; ** p < 0.01; *** p < 0.001 \|  \|  \|  \|  \|   Table 9A. Likelihood ratio tests comparing multilevel models | | | | | | |  |
| --- | --- | --- | --- | --- | --- | --- | --- | --- | --- | --- | --- | --- | --- | --- | --- | --- | --- | --- | --- | --- | --- | --- | --- | --- | --- | --- | --- | --- | --- | --- | --- | --- | --- | --- | --- | --- | --- | --- | --- | --- | --- | --- | --- | --- | --- | --- | --- | --- | --- | --- | --- | --- | --- | --- | --- | --- | --- | --- | --- | --- | --- | --- | --- | --- | --- | --- | --- | --- | --- | --- | --- | --- | --- | --- | --- | --- | --- | --- | --- | --- | --- | --- | --- | --- | --- | --- | --- | --- | --- | --- | --- | --- | --- | --- | --- | --- | --- | --- | --- | --- | --- | --- | --- | --- | --- | --- | --- | --- | --- | --- | --- | --- | --- | --- | --- | --- | --- | --- | --- | --- | --- | --- | --- | --- | --- | --- | --- | --- | --- | --- | --- | --- | --- | --- | --- | --- | --- | --- | --- | --- | --- | --- | --- | --- | --- | --- | --- | --- | --- | --- | --- | --- | --- | --- | --- | --- | --- | --- | --- | --- | --- | --- | --- | --- | --- | --- | --- | --- | --- | --- | --- | --- | --- | --- | --- | --- | --- | --- | --- | --- | --- | --- | --- | --- | --- | --- | --- | --- | --- | --- | --- | --- | --- | --- | --- | --- | --- | --- | --- | --- | --- | --- | --- | --- | --- | --- | --- | --- | --- | --- | --- | --- | --- | --- | --- | --- | --- | --- | --- | --- | --- | --- | --- | --- | --- | --- | --- | --- | --- | --- | --- | --- | --- | --- | --- | --- | --- | --- | --- | --- | --- | --- | --- | --- | --- | --- | --- | --- | --- | --- | --- | --- | --- | --- | --- | --- | --- | --- | --- | --- | --- | --- | --- | --- | --- | --- | --- | --- | --- | --- | --- | --- | --- | --- | --- | --- | --- | --- | --- | --- | --- | --- | --- | --- | --- | --- | --- | --- | --- | --- | --- | --- | --- | --- | --- | --- | --- | --- | --- | --- | --- | --- | --- | --- | --- | --- | --- | --- | --- | --- | --- | --- | --- | --- | --- | --- | --- | --- | --- | --- | --- | --- | --- | --- | --- | --- | --- | --- | --- | --- | --- | --- | --- | --- | --- | --- | --- | --- | --- | --- | --- | --- | --- | --- | --- | --- | --- | --- | --- | --- | --- | --- | --- | --- | --- | --- | --- | --- | --- | --- | --- | --- | --- | --- | --- | --- | --- | --- | --- | --- | --- | --- | --- | --- | --- | --- | --- | --- | --- | --- | --- | --- | --- | --- | --- | --- | --- | --- | --- | --- | --- | --- | --- | --- | --- | --- | --- | --- | --- | --- | --- | --- | --- | --- | --- | --- | --- | --- | --- | --- | --- | --- | --- | --- | --- | --- | --- | --- | --- | --- | --- | --- | --- | --- | --- | --- | --- | --- | --- | --- | --- | --- | --- | --- | --- | --- | --- | --- | --- | --- | --- | --- | --- | --- | --- | --- | --- | --- | --- | --- | --- | --- | --- | --- | --- | --- | --- | --- | --- | --- | --- | --- | --- | --- | --- | --- | --- | --- | --- | --- | --- | --- | --- | --- | --- | --- | --- | --- | --- | --- | --- | --- | --- | --- | --- | --- | --- | --- | --- | --- | --- | --- | --- | --- | --- | --- | --- | --- | --- | --- | --- | --- | --- | --- | --- | --- | --- | --- | --- | --- | --- | --- | --- | --- | --- | --- | --- | --- | --- | --- | --- | --- | --- | --- | --- | --- | --- | --- | --- | --- | --- | --- | --- | --- | --- | --- | --- | --- | --- | --- | --- |
|  | SL | Model 1 (VC) | Model 2 (RI) | Model 3 (RS) | Model 4 (RS) |  |  |
| *Pre-Transform* |  |  |  |  |  |  |  |
| Log-likelihood | -8929.50 | -8915.12 | -7753.72 |  |  |  |  |
| d.f. |  | 1 |  |  |  |  |  |
| LR chi2 |  | 28.76 |  |  |  |  |  |
| p |  | 0.00 |  |  |  |  |  |
|  |  |  |  |  |  |  |  |
| *Post-Transform* |  |  |  |  |  |  |  |
| Log-likelihood | -9535.78 | -9517.58 | -8436.89 | -8374.24 | -8368.31 |  |  |
| d.f. |  | 1 |  | 2 | 2 |  |  |
| LR chi2 |  | 36.38 |  | 125.31 | 11.85 |  |  |
| p |  | 0.00 |  | 0.00 | 0.00 |  |  |
